# Exploring the challenges to telephone triage in pre-hospital emergency care: a qualitative content analysis

**DOI:** 10.1186/s12913-022-08585-z

**Published:** 2022-09-23

**Authors:** Fateme Mohammadi, Ali Khani Jeihooni, Parisa Sabetsarvestani, Fozieh Abadi, Mostafa Bijani

**Affiliations:** 1 Department of Nursing, Chronic Diseases (Home Care) Research Center and Autism Spectrum Disorders Research Center, Hamadan University of Medical Sciences, Hamadan, Iran; 2 Department of General Health, Shiraz University of Medical Sciences, Shiraz, Iran; 3 School of Nursing, Fasa University of Medical Sciences, Fasa, Iran; 4 Department of Medical Surgical Nursing, Fasa University of Medical Sciences, Fasa, Iran

**Keywords:** Emergency medical service, Telephone triage, Health services, Qualitative research

## Abstract

**Background:**

One of the important indices for the efficacy of pre-hospital emergency services is telephone triage. The dispatching team members are faced with many challenges in telephone triage which can adversely affect their performance. This study was conducted in the south of Iran to determine the challenges to telephone triage in pre-hospital emergency services.

**Method:**

The present study is qualitative-descriptive where the sample was selected purposefully. Data were collected through 18 semi-structured, in-depth interviews with 18 dispatching team members in pre-hospital emergency care. The collected qualitative data were analyzed using the content analysis approach recommended by Graneheim and Lundman.

**Results:**

Analysis of the data resulted in the emergence of three themes and ten sub-themes. The three main themes extracted from the data included inefficient interaction, insufficient and unreal information, and professional challenges.

**Conclusion:**

The dispatching unit personnel in pre-hospital emergency care are confronted with various interactional, organizational, and professional issues. Accordingly, the senior managers in emergency departments should take effective measures to remove the existing barriers toward improving the efficacy of telephone triage and, by extension, the quality of pre-hospital emergency care services.

**Supplementary Information:**

The online version contains supplementary material available at 10.1186/s12913-022-08585-z.

## Introduction

In today’s world, pre-hospital emergency care is an integral part of treating patients who are in need of emergency care [1]. Pre-hospital emergency care comprises the emergency care services provided to patients outside the hospital environment and often end with the transfer of the patients to the nearest medical center [1]. In pre-hospital emergency care, seconds can mean the difference between life and a serious disability or death of the patient. Thus, timely provision of care and quick evaluation of the patients’ status by pre-hospital emergency care personnel are infinitely important [2]. The dispatching unit personnel in pre-hospital emergency care play the main role in telephone triage [3].

As the first point of contact between patients and the emergency care department, the dispatching unit gives the first direct response to requests for emergency care. The dispatching unit personnel not only assess the urgency of a call and dispatch a team accordingly, they also try to give counselling to the caller so as to minimize the consequences of the accident or disease and manage the patient/victim as well as the scene of the accident before the arrival of the emergency team [4, 5]. According to a study by Holmström et al. in Sweden, the dispatching unit personnel who were in charge of telephone triage in pre-hospital emergency care were faced with various problems, including inadequate knowledge, lack of a single standard protocol for evaluation of patients’ conditions, work overload, unsatisfactory clinical decision-making skills, and numerous telephone calls, all of which adversely affected their performance in triage [6]. The results of a study by Azarnoush et al. revealed that the skill and knowledge of the nurses who perform telephone triage in the dispatching unit have a significant impact on the decisions made by the pre-hospital emergency care personnel. Accordingly, the dispatching unit personnel were faced with various problems, including diversity of languages and cultures, inaccurate addresses, lack of proper clinical guidelines, plus occupational fatigue and stress [7]. The results of a study by Delshad et al. showed that in addition to human factors, the presence of resources and the use of digital technology including global positioning system (GPS) have a significant impact on the quality of emergency medical services. Also, applying global positioning system (GPS) is helpful and can aid in identifying the location of a call and be a valuable tool in reducing pre-hospital transport time [8].

### Background in Iran

In Iran, the dispatching unit personnel who are in charge of telephone triage and giving medial counselling in pre-hospital emergency care are nurses with a bachelor’s degree or emergency nurses with a bachelor’s degree or an associate degree. The dispatchers work in 12- or 24-hour shifts. After receiving a call, the dispatching unit personnel obtain the necessary information from the caller and give medical counselling if needed. In urgent situations, they dispatch an emergency care team to evaluate the patient’s conditions or the scene of the accident and continue to give the necessary counselling to the caller before the dispatched team arrives. In Iran the dispatching unit personnel in pre-hospital emergency care have limited digital technology, such that only a few numbers of pre-hospital centers use GPS services.

There is only a small body of knowledge about telephone triage as performed by the dispatching unit in pre-hospital emergency care as well as the influential factors and barriers which affect their performance in reality. In addition, only a few studies have addressed this area qualitatively. Indeed, the researchers’ experience of practice in pre-hospital emergency care showed that the personnel who are in charge of telephone triage are faced with many problems which adversely affect the quality of services provided by pre-hospital emergency care teams. The present study was conducted in the south of Iran to determine the challenges to telephone triage in pre-hospital emergency services.

## Methods

The design employed in this study was descriptive qualitative, which has been confirmed to be an effective method in studies carried out on the subjects’ perception about various dimensions of the concept to be studied [9]. Descriptive qualitative method is practical when researchers intend to study and recognize the participants’ perception of a phenomenon in cases for which insufficient information is available [10]. We used the checklist for consolidated criteria for reporting qualitative research (COREQ) to report the findings obtained in our study [11].

### Participants

Eighteen members of the dispatching unit who had work experience in the four emergency medical service centers in the south of Fars province, Iran, participated in the present study. They worked and had in-depth experience in triage by telephone; purposeful sampling was used to select and enroll the participants. Thus, workers with high experience or knowledge about the subject under study were recruited. We consulted the manager of the emergency medical services center to help us select one of the personnel with good communication skills who could provide us with sufficient information in the interview stage of the study. Then, we asked the first participant to name another colleague with the mentioned abilities as well as skills and continued the sampling until data saturation. In interviews, in case no new knowledge, concept, or dimensions are obtained, data saturation has been reached and data collection is halted [12].

Once 18 interviews were done, data saturation was achieved. To ensure that no new data could be obtained, two further interviews were done. At least one year wok experience in dispatching unit; lack of fatigue, as expressed by the interviewee due to work overload when the interview was done; and readiness for attending the interview sessions which lasted about 45–60 minutes constituted the inclusion criteria in this study. We excluded those who were unwilling to participate in and continue the study.

### Data collection

Semi-structured individual interviews were performed from October to February 2022. Totally, 18 interviews were performed. Prior to data collection through interviews, the interviewees were provided with sufficient information about the aim of the study, methods of data collection, reasons for recording their interviews, and the roles of the interviewer plus interviewees. They were assured that their participation was voluntary, they could withdraw from the study whenever they wished, and their data would remain confidential. Having arranged with the director of the center, we used WhatsApp, to interview the participants through video calls in the conference room of the emergency medical services center. The first author (FM) did the interviews; each interview lasted about 45–60 minutes and the researcher recorded the interviews using a Voice Recorder. The interviewer asked about challenges to telephone triage plus some probing questions such as “Can you explain more?”, “What do you mean?”, and “Can you give an example?”. This was done to enhance the clarity of data (Supplementary file: Interview Guide and Questions).

### Data analysis

Graneheim and Lundman’s content analysis method (2004) was employed to gather the qualitative data and analyze them. Based on this method, we read the transcribed interviews several times to immerse in the data and extract the complete concepts. Afterward, the semantic units and codes were verified in a session arranged with the team members. In the case of disagreement, they were resolved and decided by the members. Then, we classified, coded, and labeled the transcripts. The codes were then examined again, similar ones were merged, and the codes as well as transcripts were compared. Then, we classified the codes and developed the categories. Subsequently, the categories were double-checked to make sure they are consistent. Ultimately, the final themes were extracted and finalized [13].

Once the interviews were finished, the recorded contents were transcribed immediately by the first author (FM), with the corresponding author (MB) reviewing and verifying the semantic units plus open codes. A meeting in which all members (FM, AJ, PS, FA and MB) attended was arranged in order to resolve the disagreements, which finally ended in verification of the data analysis quality. Then, after several joint meetings, the categories were reflected on carefully by the team members, and the main themes were extracted. As reported in Table 1 (an example of the data analysis process), MAXQDA v. 2007 was used to analyze the data.

**Table 1:** An example of coding and development of categories and themes

Meaning units	Coding	Category	Theme
*”On many occasions, as soon as we pick up the phone, the caller starts to shout angrily and insult us. Yet, we politely ask them to calm down and describe their patient’s condition so we can assist them. But, unfortunately, they keep shouting and cursing and tell us to just send an ambulance right away and not ask any questions.” (Participant 5*)	Insult and anger of the caller	Distressed and rude callers	Inefficient interaction
*”Telephone triage is really stressful. Sometimes, we are flooded with phone calls and, because we have to work extended shifts, we get exhausted and can’t concentrate. The stress and fatigue at work results in early burnout in the personnel. We aren’t made of steel; we can deal with the pressure up to a threshold; when we are fatigued, our efficiency decreases.” (Participant 10)*	work extended shifts Stress and fatigue at work	Occupational fatigue and stress	Professional barriers

### Rigor

The accuracy and trustworthiness of the collected data were assessed using Guba and Lincoln’s criteria (1985). The researchers used prolonged engagement, member checking, and peer review to ensure the study’s credibility. For member-checking, a copy of the coded interviews was given to seven pre-hospital emergency care personnel who were asked to evaluate the accuracy of the data. For peer-checking, five experts observed as well as evaluated the process of data analysis and finally validated the codes plus categories. To ensure the dependability and confirmability of the categories, the researchers used audit trail, which involved application of precise interview techniques, accurate transcription, and peer review. To enhance the transferability of the findings, the researcher provided accurate descriptions of the concept under study, characteristics of the participants, method of data collection, methods used to analyze the data, as well as documented samples of the participants’ quotes [14].

## Results

Of the 18 participants, 12 were female and six were male. The means age of the participants and lengths of work experience were 37.66 ± 7.56 years old and 12.11 ± 6. 49 years, respectively. Table 2 presents the other demographic characteristics of the participants. Analysis of the qualitative data resulted in three themes and ten sub-themes. The three main themes were inefficient interaction, insufficient and unreal information, and professional barriers. The themes and sub-themes are outlined in Table 3.

**Table 2:** Individual characteristics of the participants

Variable		N (%)
Work experience	2–10	8 (44.45)
	11–15	3 (16.67)
	16–23	7 (38.88)
Age	25–30	5 (27.78)
	31–40	6 (33.34)
	41–50	7 (38.88)
Education level	Associate’s degree in EMS	4 (22.23)
	Bachelor’s degree in EMS	5 (27.77)
	Bachelor’s degree in nursing	9 (50)

**Table 3:** Themes and subthemes extracted from content analysis

Themes	Subthemes
Inefficient interaction	• Distressed and rude callers • Hoax call or Prank call • Diversity of languages and cultures
Insufficient and unreal information	• Fear of giving adequate information and an accurate account • Very young callers • Inaccurate addresses
Professional barriers	• Lack of proper clinical guidelines • Lack of professional knowledge and skills • Ineffective continuing education and personal development • Occupational fatigue and stress

### Inefficient interaction

One of the themes extracted from the participants’ experiences and views was inefficient interaction with the subcategories of distressed and rude callers, telephone harassment, as well as diversity of languages and cultures.

#### Distressed and rude callers

The dispatchers’ experiences showed that one of the major issues in telephone triage is the insulting behavior of the citizens who call and request emergency care services. The callers’ rude attitude distracts and disturbs the dispatching personnel during telephone triage with adverse effects on their decision-making and counseling. According to one of the participants:

*“On many occasions, as soon as we pick up the phone, the caller starts to shout angrily and insult us. Yet, we politely ask them to calm down and describe their patient’s condition so we can assist them. But, unfortunately, they keep shouting and cursing and tell us to just send an ambulance right away and not ask any questions.” (Participant 5)*

#### Hoax call or prank call

Another sub-theme of inefficient interaction was hoax or prank call. Indeed, the participants stated that they had many cases of hoax call or prank call every day and, on some occasions these unwanted calls had put patients’ lives at risk.

According to one of the participants:

*“This is a sociocultural issue we’ve encountered many times. Someone contacts the pre-hospital emergency center and says that they have a patient with a cardiovascular condition who needs immediate medical attention. After we dispatch a team to the given address, we realize that the request was unreal and the caller only wanted to prank call.” (Participant 7)*

Another participant stated that:

*“I have a very disturbing experience of prank and hoax call in one my shifts. Someone called and said that his brother had been in a terrible car crash and had received a severe blow to the head and he asked for an ambulance. I dispatched an ambulance to the given address. At the same time, someone else called and requested an ambulance for a patient who had cardiac arrest. Because there were a lot of missions that day, we were short of ambulances. Unfortunately, the first phone call turned out to be a fake request and the life of the second patient with cardiac arrest was jeopardized because there were no ambulances and he had to be taken to the hospital in a car. Sadly, that patient lost his life.” (Participant 4)*

#### Diversity of languages and cultures

Sometimes, the telephone triage personnel in the dispatching unit encounter a diversity of languages and cultures. Unfamiliarity with a caller’s accent, dialect, or language can prevent them from providing the necessary counselling and information to the caller, which adversely affects effective provision of emergency care services. According to one of the participants:

*“Occasionally, I’ve received calls from people who spoke in a certain dialect or language and I couldn’t understand what they wanted. In one of my shifts, someone called the emergency care center and spoke in Arabic. I had no idea what he was saying. Because I didn’t know Arabic, I couldn’t help him.” (Participant 12)*

### Insufficient and unreal information

Another theme extracted from the data was insufficient and unreal information, which was comprised of the following sub-categories: fear of giving adequate information and an accurate account, very young callers, and inaccurate addresses.

#### Fear of giving adequate information and an accurate account

Based on the participants’ experiences, in some cases, callers refuse to provide adequate and accurate information for fear of stigma or having performed culturally and socially inappropriate behaviors. One of the participants stated that:

*“In one of my shifts in the dispatching unit, someone called and said her daughter had swallowed 10 tranquilizers. I asked if she’d had anything else and the caller said no. When the emergency care team transferred her to the hospital, they realized she’d drunk alcoholic drinks and her parents had hidden the truth because they were worried about the social stigma of drinking, so they gave the dispatching unit inaccurate information”. (Participant 15)*

One of the participants stated that:

*“On many occasions, patients have been sexually assaulted, but due to sense of shame and fear of social stigma, the patients do not provide accurate and complete information and emergency medical personnel make mistakes in diagnosing the patients' problems”. (Participant 13)*

#### Very young callers

Another sub-theme of insufficient and unreal information was receiving calls from children. The participants’ experiences showed that occasionally children call the emergency care centers and request help. Since children are not able to provide sufficient and accurate information to dispatchers, they may get confused and fail to determine the patients’ basic problem; consequently, their services will not be adequately effective. According to one of the participants:

*“We’ve encountered this problem many times: a very young child or old person calls the dispatching unit and asks for an ambulance, but when we ask, “What is your emergency?” they can’t give enough information. They just say the patient’s condition is not good and they don’t know what to do and then hang up. When the emergency care personnel arrive at the scene, they realize the patient’s had cardiac arrest.” (Participant 18)*

#### Inaccurate addresses

According to the participants’ experiences, one of the major barriers to providing effective pre-hospital emergency care services is callers’ failure to provide an accurate address. Giving care in the quickest time possible is essential in the quality and efficacy of pre-hospital emergency care services. However, sometimes callers fail to give a complete or accurate address, and, since the dispatching units have to use the information provided by callers, the inaccuracy of that information results in delay in the provision of medical services to patients. In several cases, patients’ lives had been put at risk since the emergency care team did not arrive as early as needed. According to one of the participants:

*“A few times, the ambulance personnel complained to the dispatchers since they hadn’t given them the right address and they were lost and it took them a long time to find the patients’ location. They are right to complain, but we dispatch ambulances based on the information and addresses the callers give us. The problem is with the incorrect information the callers provide.” (Participant 8)*

### Professional barriers

The final theme extracted from the collected data is professional barriers, which consists of the sub-themes of lack of proper clinical guidelines, lack of professional knowledge and skills, ineffective continuing education and personal development, and occupational fatigue and stress.

#### Lack of proper clinical guidelines

The participants’ experiences showed that one of the major barriers to telephone triage in dispatching units is lack of proper and comprehensive clinical guidelines. The participants stated that since there was not a set of fixed clinical guidelines, they got confused at the time of telephone triage and could not give satisfactory counselling as a result. According to one of the participants:

*“We lack a single set of complete clinical guidelines for telephone triage. Some of the existing guidelines are ambiguous. One manual says there is no need to dispatch an ambulance; another says you must send an ambulance. We really don’t know what to do and which instructions to follow. The national department of emergency care services should really develop a single set of comprehensive protocols for the dispatchers.” (Participant 11)*

#### Lack of professional knowledge and skills

Based on the participants’ experiences, one of the main issues in telephone triage is the dispatchers’ inadequate professional knowledge and experience as well as inexperienced personnel. The participants stated that if the dispatching unit personnel do not have the required professional knowledge and experience for telephone triage, they may not be able to give the right counselling in life-threatening situations, e.g. airway obstruction or cardiac arrest, and may even put a patient’s life at risk by giving the wrong information to the caller. According to one of the participants:

*“Some of my colleagues here in the telephone triage unit don’t have enough clinical knowledge and experience. Some of them don’t know anything about urgent or life-threatening emergencies. Their clinical knowledge about diagnosing and treating diseases is not up to date. In my opinion, telephone triage is a very difficult and sensitive job and only a few are qualified enough to work in this unit. To execute successful telephone triage, you need to be astute and possess adequate academic knowledge as well as clinical and professional experience so you can determine the patient’s problem within the shortest time by asking a few key questions and giving accurate counselling before an ambulance arrives at the patient’s location.” (Participant 9)*

Another participant stated that:

*“I remember once a person called the pre-hospital emergency care center and said that her mother had a severe pain in her stomach and she asked the dispatcher to send an ambulance. My colleague was not experienced enough in telephone triage and told the caller that stomach pain did not count as an emergency and there was no need for an ambulance. Unfortunately, that patient had cardiac arrest and they sued the emergency department.” (Participant14)*

#### Ineffective continuing education and personal development

From the participants’ point of view, successful telephone triage depends on the active participation of pre-hospital emergency care dispatchers in continuing education and personal development programs, so that they can update their academic knowledge as well as awareness of clinical guidelines to give effective counselling to callers. Thus, senior managers in emergency medical care departments are recommended to take measures in order to ensure continuing personal development of the personnel in dispatching units. According to one of the participants:

*“There aren’t enough workshops on telephone triage. They may hold just one workshop every year and some of the personnel can’t even attend that due to their extended shifts. To keep the telephone triage personnel’s knowledge up to date, we need continuing educational programs which target the personnel’s personal and professional development.” (Participant 6)*

#### Occupational fatigue and stress

The participants’ experiences indicated that the telephone triage in dispatching units is a very demanding and stressful job. The extended shifts and job fatigue due to work overload lead to occupational stress as well as job burnout in the personnel.

According to one of the participants:

*“Telephone triage is really stressful. Sometimes, we are flooded with phone calls and, because we have to work extended shifts, we get exhausted and can’t concentrate. The stress and fatigue at work results in early burnout in the personnel. We aren’t made of steel; we can deal with the pressure up to a threshold; when we are fatigued, our efficiency decreases.” (Participant 10)*

## Discussion

This study was conducted in the south of Iran to determine the challenges to telephone triage in pre-hospital emergency care services and to suggest effective solutions for them. Analysis of the experiences of the dispatchers who were in charge of telephone triage resulted in three themes: inefficient interaction, insufficient and unreal information, and professional challenges. One of the main barriers to successful telephone triage was inefficient interaction, which was caused by distressed and rude callers, hoax call or prank call, as well as diversity of languages and cultures. During telephone triage, the dispatching unit personnel sometimes encounter hostile callers who demand an ambulance in an insulting manner. Hoax call or prank call as well as variations in languages and cultures are other significant issues under the theme of inefficient interaction. Similarly, the findings of a study by Holmström et al. in Sweden showed that the dispatchers in pre-hospital emergency care services could not effectively communicate with callers who were agitated and aggressive at the time of telephone triage. It was also found that the high frequency of telephone calls as ell as false requests and hoax call or prank call had adverse effects on the quality of telephone triage. Another obstacle to telephone triage in this study was language barriers: more than half of the calls the dispatching unit received were in a language other than Swedish [6]. On a similar note, Montandon et al. as well as Meischke et al. reported language problems as one of the major obstacles to pre-hospital telephone triage. When dispatchers and callers do not speak the same language, the former cannot communicate effectively and determine the patients’ basic problems [15, 16].

The results of a study Furaijat et al. in Germany as well as Noacket al. in Mexico showed that the use of digital communication assistance tool (DCAT) for overcoming language barriers can be a practical method when providing health care to foreign language patients [17, 18]. The findings of a study by Alm-Pfrunder et al. in Sweden showed that the prehospital emergency care nurses’.

used to the body language and tone of voice for creating a sense of trust and security while assessing and caring for patients when there was no mutual language. Furthermore, translation equipment and relatives/bystanders were used as interpreters when possible [19]. Thus, the senior managers in emergency departments in Iran should take measures to use digital communication assistance tool (DCAT) to overcome language barriers.

Another theme extracted from the data collected in the present study was insufficient and unreal information with the sub-categories of fear of giving adequate information and an accurate account, very young callers, and inaccurate addresses. According to a study by Møller et al. along with Ek et al., when they received a call, the pre-hospital emergency staff were sometimes given incomplete or inaccurate addresses. In addition, when the callers were very young children or old people who suffered from dementia, they could not give an accurate account of their patients’ status, which confused the personnel [20, 21]. Accordingly, healthcare policymakers and administrators should organize awareness campaigns to educate the public about using pre-hospital emergency services, so that they can make effective and efficient use of those services.

The final theme extracted in the present study was professional barriers, comprised of lack of proper clinical guidelines, lack of professional knowledge and skills, ineffective continuing education and personal development, and occupational fatigue and stress. Palma et al. along with Sakurai et al. similarly found that lack of comprehensive and practical clinical protocols as well as execution of telephone triage by inexperienced personnel who did not have the required professional knowledge had significant adverse effects on the quality of pre-hospital and hospital emergency care services. The results of this study also revealed that use of personnel with adequate experience and knowledge of telephone triage was influential in reducing the number of non-urgent missions: accurate telephone triage and good counselling can eliminate the need for many pre-hospital urgent missions and dispatch of ambulances [22, 23]. In a similar study, Bijani et al. reported that inexperienced personnel who did not possess adequate professional knowledge and competence adversely affected the clinical decisions made by the pre-hospital emergency care personnel [24]. The results of the present study also indicated that work overload, extended shifts, and lack of regular educational programs designed to enhance the personal and professional development of the personnel were other significant barriers to provision of high-quality pre-hospital emergency care services. Thus, emergency care mangers should take the necessary steps to develop practical clinical guidelines, improve the personal and professional development of the personnel, and eliminate professional obstacles, including occupational stress and job burnout, to enhance the effectiveness and efficiency of emergency services. Accordingly, to improve the quality and efficacy of emergency medical services, the authorities should take measures to eliminate the professional barriers, including stress and fatigue. The findings of a study by Smith et al. showed that the dispatchers in pre-hospital emergency care services experience both physical and mental health challenges as a result of their work, including compassion fatigue, occupational burnout, emotional exhaustion, inadequate debriefing after stressful calls, lack of employee training for mental-health-related calls, and exposure to verbally aggressive callers [25].

### Limitations

In this study, the researchers only explored the experiences and views of the dispatches in pre-hospital emergency care regarding telephone triage. It is suggested that future research addresses the views of the other stakeholders including system administrators, patients, and the beneficiaries of pre-hospital emergency care services. However, in view of the economic, cultural, and social differences between Iran and other countries, it is recommended that similar studies should also be conducted in other countries. Studies should also be performed to address the challenges of telephone triage.

### Strengths

The present study is the first qualitative attempt at identifying the barriers to pre-hospital telephone triage for dealing with them in the south of Iran, which makes it an innovative work of research.

## Conclusion

With regard to telephone triage, the dispatching personnel in pre-hospital emergency care are faced with various interactional, organizational, and professional issues. Accordingly, the senior managers in emergency departments should take effective measures and provide support to remove the existing barriers. Development of clinical guidelines, employment of experienced personnel (with adequate knowledge and professional competence), programs designed to enhance the personal development of the personnel, elimination of the causes of occupational stress and job burnout, and enhancement of public awareness about how to use pre-hospital emergency services will contribute to the efficacy of telephone triage which will, in turn, improve the quality of pre-hospital emergency medical services.

## Supplementary Information

**Additional file 1.** 

## Data Availability

The datasets generated and/or analysed during the current study are not publicly available due to the necessity to ensure participant confidentiality policies and laws of the country but are available from the corresponding author on reasonable request.

## Abbreviations

GPS: Global positioning system
EMS: Emergency Medical Services
DCAT: Digital communication assistance tool
