# Supplementary material for: Exploring the challenges to telephone triage in pre-hospital emergency care: a qualitative content analysis
Source: BMC Health Serv Res. 2022 Sep 23;22:1195. doi: 10.1186/s12913-022-08585-z (PMC9502955; doi:10.1186/s12913-022-08585-z)
Supplement: Supplementary file 1 — Additional file 1. [file 12913_2022_8585_MOESM1_ESM.docx]

| ***Supplementary file : Interview Guide and Question*** |
| --- |
| **Interview Guide**  Thank you for accepting to be interviewed by us. The study we are undertaking is to understand more about the “***Exploring the challenges to telephone triage in pre-hospital emergency care: a qualitative content analysis***  ”. I will be asking you several questions which are relevant to the study. You may respond to these queries in any way you feel comfortable. It is perfectly fine if you do not want to respond. At any point during the interview, if you are not clear about any questions, you are free to clarify the same with us and ask us to explain further. The information obtained during the interview will be kept confidential and will be shared only with the research team. We would like to audio record the interview in order to ensure that we do not miss out any salient issues. The recordings will be kept confidential. Your identity will be protected and your interview will also be labeled in codes. Is it OK with you that we audio record the interview?  **Interview questions**:   1. “Can you describe your typical work day in the dispatching unit and experience of telephone triage?” 2. What challenges are pre-hospital emergency care personnel to telephone triage 3. “What factors can affect your decisions during telephone triage?” 4. “Do your colleagues have the required skills for telephone triage?” |
